# Dormant *Pseudomonas aeruginosa* infection seven years post-augmentation mastopexy: A case report

**DOI:** 10.1016/j.ijscr.2021.106614

**Published:** 2021-11-19

**Authors:** Mamoon Daghistani, Maha Hanawi, Nouf Alturki

**Affiliations:** a Department of Plastic Surgery, King Abdulaziz Medical City, Jeddah, Saudi Arabia; b College of Medicine, King Saud bin Abdulaziz University for Health Sciences, Jeddah, Saudi Arabia

**Keywords:** Breast implant, Capsular contracture, Idiopathic, Late-onset, *pseudomonas aeruginosa*, Mastopexy

## Abstract

**Introduction and importance:**

Around 1% of all complications associated with breast implants are attributable to infection, classified as acute, subacute, or late-onset, with late-onset infections being the rarest. Even when symptoms are not obvious, an infection may still be lingering. Sub-clinical presentations have been implicated in the pathophysiology of breast implant capsular contracture. Organisms can establish dormancy through biofilm formation, and can also be idiopathically activated, and present as a late-onset infection, as has been clearly described in the literature with the infamous *Enterococcus avium*.

**Case presentation:**

We report the case of a 44-year-old woman who underwent bilateral augmentation mastopexy seven years ago complicated by an acute perioperative infection that was resolved with a full course of antibiotics. She presented to the clinic complaining of left breast pain and swelling accompanied by fever for four days. Ultrasonic imaging showed moderate peri-implant fluid positive for *Pseudomonas aeruginosa* upon aspiration. The patient therefore underwent bilateral breast exploration and capsulectomy.

**Clinical discussion:**

We believe that the dormant *P. aeruginosa* contributed to the capsular contracture and was idiopathically activated, manifesting as a late-onset infection seven years post-augmentation mastopexy.

**Conclusion:**

To the best of our knowledge, no previous studies or case reports have described a late-onset infection due to idiopathic activation, where dormant *P. aeruginosa* is isolated from an implant capsule many years after augmentation mastopexy. More studies are required to examine the role of dormant bacteria in capsular contracture and their idiopathic activation considering the consequences on patient outcomes.

## Highlights

• Capsular contracture is the commonest complication after breast implantation.
• Subclinical infections are implicated in capsular contracture pathogenesis.
• *Pseudomonas aeuroginosa* may cause acute-onset and late-onset infections.
• Biofilm formation helps bacteria establish dormancy in infected tissues.
• Dormant bacteria cause capsular contracture and are idiopathically activated.

## 1 Introduction

Breast augmentation is one of the most performed procedures in plastic surgery. Prosthetic implants are often used to augment breast size, although fat grafting is also utilized for the same purpose, but to a lesser extent [1]. Implants are classified as smooth or textured based on the surface texture; however, the latter are less preferred as they appear to increase the risk of breast implant-associated anaplastic large cell lymphoma (BIA-ALCL), a rare but serious complication. Indeed, a patient with textured-type breast implants who presents with seroma within a year or more of implantation has a 10% risk of BIA-ALCL. Therefore, any patient suspected of having BIA-ALCL should undergo immunohistochemistry for CD30 and anaplastic lymphoma kinase (ALK) [2]. Complications after breast implants include hematoma, infections, breast asymmetry, rippling, and capsular contracture. Obesity, tobacco smoking, and comorbid diseases may increase the risk of developing such complications postoperatively [1], [2], [3], [4].

Infection after breast implants has a prevalence rate of 0.74%, subdivided into acute, subacute, and late infections [4]. Acute infections occur in the first six weeks postoperatively, and Gram-positive cocci are the most commonly isolated organisms, followed by Gram-negative bacilli. Conversely, in subacute infections, which occur between six weeks and six months postoperatively, staphylococci and Gram-positive anaerobic bacilli are typically the culprits. Any infection occurring after six months is considered late-onset, and the hematogenous seeding of bacteria accounts for most cases [3], [4]. Subclinical infections have been implicated in the pathogenesis of capsular contracture [5]. This work has been reported in line with the SCARE criteria [6].

## 2 Presentation of case

A 44-year-old female patient with a history of heavy tobacco smoking who underwent bilateral augmentation mastopexy seven years ago presented to the clinic complaining of left breast pain, swelling, and firmness lasting for four days, accompanied by fever. The patient was alert and vitally stable. On physical examination, left-sided breast swelling, rigidity, and tenderness were noted. No masses or changes in temperature were observed. Contralateral breast examination findings were unremarkable.

The patient had a body mass index of 20.29 kg/m^2^, was known to suffer from bipolar II disease, major depression, and irritable bowel syndrome (IBS), which were controlled on medications: quetiapine, citalopram, zolpidem, and lamotrigine. The patient's family history was unremarkable.

In 2014, the patient underwent bilateral augmentation mastopexy using silicone gel-textured implants in the subfascial plane through inframammary incisions. The surgery was performed by a senior plastic surgeon, Dr. Mamoon. The patient tolerated the procedure well and was discharged when stable. However, six days after the procedure, the patient developed bilateral breast seroma. Due to possible superficial infections at the incision sites, empirical antibiotics were started, and the seroma in both breasts was evacuated. The fluid was sent for culture, and the patient was discharged with a ciprofloxacin and clindamycin prescription for 10 days. Breast seroma culture revealed *P. aeruginosa* isolated in broth media that was sensitive to both antibiotics. At the first postoperative clinic visit, she had completed her antibiotic course, and her postoperative condition was uneventful.

**Fig. 1:**
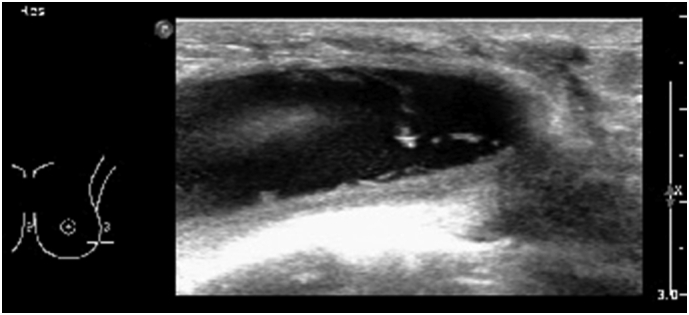
Left breast ultrasound depicting a thick wall of left breast with moderate amount of peri-implant fluid.

**Fig. 2:**
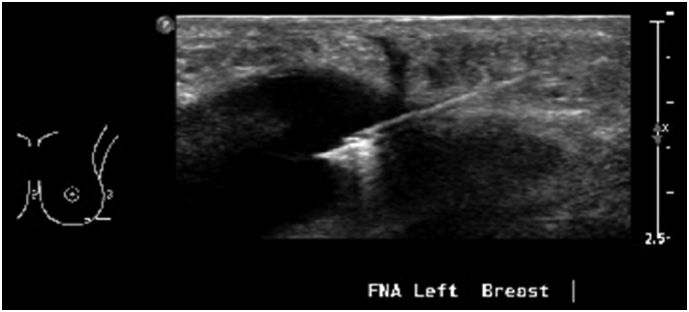
Fine needle aspiration of the peri-implant fluid of left breast for cytology and culture.

Seven years after the procedure, the patient presented to the emergency department complaining of left breast pain and swelling and received a diagnosis of left-sided Baker 4 capsular contracture and was scheduled for a follow-up in the plastics clinic. One month later, she presented to a primary healthcare clinic with a subjective fever lasting for four days and left breast pain, swelling, and tenderness. An emergency breast ultrasound of left breast revealed a moderate amount of peri-implant fluid with a thick wall, suspected to be due to extracapsular rupture (Fig. 1). Fluid aspiration was performed and samples were sent for cytology and culture (Fig. 2). Cytology showed 11% lymphocytes, mainly mature T-cells with no aberrant loss or expression of T-cell markers or decreased CD4/CD8 ratio, 80% granulocytes, 11% natural killer cells, and no B-cells. Culture revealed *P. aeruginosa* growth.

Bilateral breast exploration and capsulectomy were performed by Dr. Mamoon. The right breast capsule was normal, while the left breast capsule was thickened with peri-implant fluid. Flow cytometry and cytology were ordered to rule out BIA-ALCL, and the capsule was sent for culture and histopathological examination. The samples were negative for CD30 and ALK. Culture was positive for *P. aeruginosa*. The pathology report confirmed focal skeletal muscle fibers with no acute inflammatory cells or atypia, and left breast capsule pathology showed acute inflammatory cells, marked neutrophilic infiltration, lymphocytes, few eosinophils, granulation tissue, and intraductal papilloma. All results supported inflammation and excluded malignancy. The patient's condition improved and no signs of complications were seen at follow up.

## 3 Discussion

Capsular contracture is the most common complication following breast implantation surgery, with an incidence rate between 2.8% and 20.4%, and can cause implant rupture, necessitating its removal [4], [7]. Multiple patient-related risk factors for contracture exist, including old age, smoking, and long follow-up intervals [8]. Our patient was 37 years of age at the time of surgery, which is not considered high-risk [9]. She was a heavy smoker, which is significantly associated with capsular contracture [10]. Furthermore, the risk of developing contracture is higher in the first year post-surgery; hence, longer follow-up intervals are associated with a higher risk [11]. The patient developed a subcapsular hematoma, which has also been associated with a higher risk of contracture [12]. There are multiple theories for the pathogenesis of contracture, such as involving post-implant infections, especially subclinical infections, where biofilm formation is an important mechanism [13].

Although a late-onset infection is mainly caused by hematogenous seeding of bacteria, the delay to infection onset varies greatly in the literature, ranging from 8 months to 40 years after implant surgery [3], [4], [13]. Hematogenous seeding results from bacteremia stemming from a variety of infectious foci and is associated with different organisms. The reported sources include dental infections following invasive procedures, eye stye or bacterial stomatitis, and peritonitis following breast implantation surgery [14], [15], [16]. Unlike our case, most previous studies report an infectious origin; however, a single case study reported recurrent late implant infections related to *Enterococcus avium* with no identifiable origin [17]. With this report, we add to the literature another case of late-onset *P. aeruginosa* infection seven years after implant placement, which is around the mean onset of infection, with no clear etiology.

*P. aeruginosa* may cause both acute and late-onset infections; however, the patient was affected by both in this case. It is possible that the bacteria present at discharge had formed biofilms around the implants, keeping them in a dormant state and causing low-grade subclinical infections. This infection was enough to cause a contracture, although not enough to manifest clinically. However, seven years later, the bacteria were idiopathically activated. Organisms have been reported to gain entry to the implant in some cases [13], [15], and *P. aeruginosa* can form biofilms, allowing it to lay dormant in the tissue and protect against antibiotics, consequently inducing chronic inflammation which causes capsular contracture. Cases of capsular contracture related to subclinical *Staphylococcus epidermidis* infections have been described. However, *P. aeruginosa* has similar clinical characteristics and can be idiopathically reactivated [13].

In this case, the patient had an unremarkable history of recent infections, use of immunosuppressive medications, trauma, invasive procedures, or contiguous dermatitis, all of which have been associated with introducing *P. aeruginosa* into implants or reactivation [14]. Besides infection, there are numerous risk factors for late-onset implant infections, such as the silicone gel-filled implants used in our patient, which have been associated with a higher risk of developing an infection in a previous study [18]. Additionally, rough-textured implants provide more surface area for bacterial attachment and biofilm formation, representing potential habitats for biofilm-forming organisms [19].

## 4 Conclusion

Capsular contracture is the most common complication following breast implant surgery and has been increasingly associated with subclinical infections. Therefore, it has been suggested that the true incidence of late-onset infection is higher than that reported due to biofilm formation, rendering it undetectable on culture [2].

To the best of our knowledge, no previous studies or case reports have described a late-onset infection due to idiopathic activation where dormant *P. aeruginosa* is isolated from an implant capsule many years following augmentation mastopexy. More studies are required to examine the role of dormant bacteria in capsular contracture and their idiopathic activation considering its consequences on patient outcomes.

## Provenance and peer review

Not commissioned, externally peer reviewed.

## Funding

None.

## Declaration of competing interest

No conflict of interest.
